# Feasibility and Acceptability of Social Prescribing for Cancer Survivors

**DOI:** 10.3390/curroncol32030129

**Published:** 2025-02-25

**Authors:** Deirdre Connolly, Chloe O’Hara, Catherine O’Brien, Adrienne Dempsey

**Affiliations:** 1 Discipline of Occupational Therapy, School of Medicine, Trinity College Dublin, D08 W9RT Dublin, Ireland; chohara@tcd.ie; 2 Trinity St. James’ Cancer Institute, St. James’ Hospital, D08 NHY1 Dublin, Ireland; caobrien@stjames.ie; 3 St. James’ Hospital, D08 NHY1 Dublin, Ireland; 4 Fatima Family Resource Centre, D08 DKP6 Dublin, Ireland; adrienne@fgu.ie

**Keywords:** cancer survivors, social prescribing, physical, mental and social health, community-based activities and services, feasibility and acceptability

## Abstract

Following cancer treatment, individuals experience a range of physical, mental and social health difficulties that interfere with their ability to resume participation in pre-cancer activities. In Ireland, the National Cancer Strategy recommends community-based services to address post-treatment difficulties. Social prescribing is a community-based, non-medical service that links individuals with health-related activities and supports in their community. This study explored the feasibility and acceptability of social prescribing for cancer survivors. A mixed methods study was undertaken with individuals who had completed curative treatment for any cancer type. Recruitment was carried out in a national cancer centre. Quantitative outcomes included feasibility metrics (recruitment, intervention adherence and retention), the Frenchay Activities Index (FAI), the Hospital Depression and Anxiety Scale (HADS), the Multidimensional Assessment of Fatigue (MAF), and EORTC QLQ-C30. Qualitative interviews explored acceptability of social prescribing. Data were analysed using descriptive statistics (quantitative data) and content analysis (qualitative data). Out of 131 individuals identified as eligible to participate, 43 agreed to participate (32.8% recruitment) and 27 met a link worker and were connected to a local activity (62.7% adherence) and completed follow-up outcome measures (62.7% retention). Improvements were observed in all health-related outcomes and those interviewed identified the intervention as acceptable. Study participants attended a range of community-based activities as a result of link worker support. They also reported increased confidence, improved mental health and reduction in fatigue following attendance at community-based activities. The findings of this study indicate that social prescribing is a feasible and acceptable community-based intervention to improve the physical, mental and social health of individuals living with and beyond cancer. A pilot randomised trial is indicated to inform a definitive intervention trial.

## 1. Introduction

Social prescribing is a community-based service that supports individuals with health and social care needs to access activities, services and supports in their community [1]. It enables healthcare professionals to refer individuals with chronic diseases to a link worker, who has knowledge and links to voluntary and third-sector organisations that can meet the needs of these individuals in their local community [2]. Social prescribing has emerged as a mechanism to assist burdened healthcare systems by stimulating the growth of stronger links between primary healthcare services and voluntary sector support [1].

A variety of social prescribing models have been recorded in the literature; some initiatives are provided by the voluntary sector in community centres, others are provided by health service commissioners in GP practices and others represent a partnership between the primary health care services (GPS and other primary care staff such as nurses and occupational therapists) and voluntary sectors in community well-being hubs [3]. Despite the variety of available models, the process of social prescribing has similarities across different services [2]. A social prescribing link worker (also referred to as community navigator, coordinator or connector) meets with an individual to establish their support needs or activity preferences. The link worker identifies opportunities within the individual’s community to engage in preferred activities and supports the individual in attending the activity, including accompanying the individual to their chosen activity, as required [4]. Although different models are used to meet the needs of all service users, this often leads to difficulty in comparing outcomes of social prescribing [5]. This therefore reduces the opportunity to compare outcomes of social prescribing across different models and to make recommendations for optimal models of delivery.

There are a wide variety of community-based activities to which individuals may be linked through social prescribing, including exercise and arts-based activities, counselling, computer/technology classes, green activities/ecotherapy, volunteering, housing support and self-help/peer support groups [2]. There is emerging evidence that social prescribing may provide a range of health and well-being benefits and improved quality of life [6,7,8]; however, there is limited evidence on the impact of social prescribing on the health and well-being of cancer survivors.

Cancer survivors experience persistent symptoms such as pain, fatigue, anxiety, depression and cognitive difficulties following treatment [9]. These symptoms can impact on the performance of daily activities, thus preventing individuals from engaging in activities of importance to them [10]. This can affect quality of life and the overall health and well-being of cancer survivors [9]. With economic and time constraints affecting the provision of post-treatment services, it is becoming difficult for hospital-based services to meet the needs of individuals following completion of cancer treatment [11]. Given the potential health benefits identified for social prescribing, it is possible that this community based-support service may benefit cancer survivors.

There is a lack of published studies within the current literature that has explored the impact of social prescribing for individuals living with and beyond cancer. However, in the UK an evaluation report by Macmillan suggested that social prescribing could support cancer survivors to access community-based activities and services that may be beneficial to their health and well-being [12]. However, further research is recommended to determine the potential impact of social prescribing for individuals living with and beyond cancer and to gather the perspectives of social prescribing users. The overall aim of this study therefore was to establish the feasibility of social prescribing as a community-based intervention to improve the mental, physical and social health of individuals living with and beyond cancer. It specifically examined recruitment and retention of cancer survivors in a social prescribing intervention, acceptability of social prescribing for individuals with cancer and explored changes in outcome measures to identify potential benefits of a social prescribing intervention.

## 2. Materials and Methods

### 2.1. Procedures

Although many different social prescribing models exist, in this study the social prescribing service was located in a community centre. Following completion of baseline measures, all participants met with a social prescribing link worker to identify their health concerns and preferences for attending a local community-based service/activity. This meeting typically lasted up to 60 min. Following this initial meeting, the link worker researched where in the participant’s local community this service/activity was available. The link worker then contacted the service/activity to obtain information on the times the service/activity was available, cost of attending the service/activity, mobility requirements for attending the service/activity, etc. When they had gathered all the information about the service/activity, they contacted the participant to provide them with all the relevant information. If necessary, the link worker offered to accompany participants to the service/activity. None of the participants chose this option. The link worker then followed up with each participant two–three weeks later to enquire if they were attending the service/activity, if it was meeting their health-related needs and if they required any further support from the link worker. If no further support was required, the participant was discharged from the service.

### 2.2. Study Design

The design for this study was a convergent parallel mixed methods design which involves collecting and analysing quantitative and qualitative data simultaneously and integrating the findings to provide a comprehensive understanding of a research problem [13]. As social prescribing is considered a complex intervention with both quantifiable and nuanced outcomes, a mixed methods approach to the evaluation of social prescribing is recommended [14]. The quantitative phase measured recruitment; retention; and self-reported physical, mental and social health outcomes before and after attendance at a social prescribing service. The qualitative phase explored participants’ acceptability and experiences of social prescribing. Ethical approval was provided by the ethics committee of the recruiting hospital (2018/06 Chairman’s Action 7).

### 2.3. Sample

Participants for this study were recruited from the outpatient oncology clinics of a national cancer hospital in Ireland. The eligibility criteria for this study were individuals attending this national cancer hospital, with any cancer diagnosis, over 18 years and who had completed cancer treatment with curative intent within the past five years. Exclusion criteria included individuals with metastatic disease.

### 2.4. Recruitment Process

Members of the oncology team reviewed the medical charts of individuals attending out-patient oncology clinics and day wards over a six-month period. A leaflet was attached to the charts of those who met the inclusion criteria. When these individuals arrived for their clinic appointment, they were provided with an information leaflet and were invited to contact a member of the study team, as per the contact details provided on the study information leaflet if they were interested in receiving further details and/or wished to participate in this study. Members of the research team were also present at outpatient clinics to answer questions from potential participants. Following agreement to participate in this study, a meeting was arranged between a member of the research team and the study participant to obtain informed consent and complete the study measures. Study participants were given the option of having this meeting in person or by phone. In-person meetings were carried out in the recruiting hospital.

### 2.5. Data Collection Methods

All study measures were completed through an interview format. A member of the research team administered all the study questionnaires with study participants.

#### Study Measurements

A study-specific questionnaire was designed to collect demographic and health-related information. Demographic information included gender, age, marital status, living situation, education and employment status. Health-related information included cancer type, time since diagnosis and treatment type. Participants were also asked if they were attending any community-based activities prior to the commencement of this study.

The Hospital Anxiety and Depression Scale (HADS) is a 14-item self-reported measure that assesses levels of anxiety and depression for use in a non-psychiatric outpatient setting [15]. It consists of individual subscales for anxiety and depression with each item rated on a 4-point scale (0 = not at all, 3 = yes definitely), with a maximum total score of 21 for each subscale. Higher scores indicate higher distress. This measure has been validated with a variety of cancer populations [16].

EORTC QLQ-C30 is a self-reported questionnaire that measures the impact of cancer and treatment on an individual’s quality of life [17]. It has strong psychometric properties relevant to different cancer patient populations [18]. QLQ-C30 includes fifteen subscales grouped into three domains: Global Quality of Life, Functional Quality of Life and symptoms.

The Frenchay Activities Index (FAI) measures frequency of engagement in community, social and instrumental activities of daily living [19]. It consists of three subscales: domestic, leisure/work and outdoor activities. Scores range from 0 to 45 with higher scores indicating greater activity engagement. The FAI has strong internal consistency (α = 0.83), criterion and construct validity and test–retest reliability (r = 0.96) [19].

Multidimensional Assessment of Fatigue (MAF) is a 16-item self-reported measure of fatigue across four dimensions: severity, distress, frequency and impact on activities of daily living [20]. Additionally, it includes a Global Fatigue Index. Higher scores indicate greater fatigue. The MAF is suitable for individuals with cancer-related fatigue and has strong reliability and validity [21].

Baseline quantitative data were collected at the initial meeting between the study participant and a member of the research team before they met with the link worker (Time 0). Follow-up study measures were completed with a member of the research team ten weeks following participants’ first meeting with the social prescribing link worker (Time 1).

Qualitative data were collected through semi-structured interviews. Individual interviews are the most frequently used data collection method in qualitative research [22]. An interview guide (Supplementary File S1) asked participants to identify activities they attended in their community following their meeting/s with the link worker and if/how these activities impacted on their physical, mental and/or social health. They were also asked about their experience of the social prescribing service, if they believed social prescribing would be a helpful service for cancer survivors and if they had any recommendations to improve the service.

### 2.6. Data Analysis

Quantitative data were analysed using SPSS 22, the statistical package for social science programme (SPSS, Inc., Chicago, IL, USA). Descriptive statistics (means (SD), medians (IQR) and frequencies) were used to describe participants’ demographic data. Inferential statistics were used to analyse differences in health-related outcome measures. Due to the small sample size, a non-parametric Wilcoxon Signed Rank Test was used to assess differences in health outcomes between baseline (T0) and ten weeks following participants’ first meeting with the link worker (T1) [23].

Qualitative data were analysed using a content analysis approach to identify commonalities and differences in participants’ experiences and perceptions of social prescribing. Neergaard et al. [24] recommended a structured approach to the analysis of qualitative data for the purposes of intervention development. Within a content analysis framework, a deductive and inductive approach was used to compare the findings of this study to previously reported research on social prescribing (deductive analysis) and to identify new findings related to social prescribing for cancer survivors (inductive analysis) [25].

### 2.7. Study Rigor

Rigor of the qualitative phase of this study was ensured through Lincoln and Guba’s criteria for credibility, transferability, dependability and confirmability [26]. Credibility ensures the truth of the findings, while transferability focuses on the applicability of findings to other contexts. Dependability and confirmability emphasise the consistency and objectivity of the data [26]. Strategies used to achieve rigor in this study included member checking, where study participants were requested to review their interview scripts for accuracy, and triangulation through using quantitative and qualitative methods to confirm findings. An audit trail was maintained by the research team to document all research decisions and processes for transparency.

## 3. Results

### 3.1. Recruitment, Adherence and Retention

Over a six-month period, 563 charts of individuals attending out-patient oncology clinics and day wards were reviewed for eligibility criteria. Of the 563 individuals, 131 were identified as eligible and informed of this study. Forty-three individuals agreed to participate in this study, giving a 32.8% recruitment rate.

All 43 participants completed baseline assessments. Twenty-seven of these met with the link worker and attended community-based activities and/or services, which is a 62.7% adherence rate to this social prescribing intervention. All twenty-seven individuals completed follow-up study measures (62.7% retention). Reasons for dropouts included a change in health status (*n* = 7), unable to be contacted (*n* = 6), two people passed away and one participant had a family bereavement.

### 3.2. Demographic and Clinical Profile

The majority of participants were female (*n* = 19, 70%). The mean age was 57 years (SD 16.4). Most participants (*n* = 15, 56%) were living with at least one family member, while the remainder (*n* = 12, 44%) lived alone. Thirteen participants (48%) were married. Twelve participants (44.4%) had third-level education and nineteen (70.3%) were not working at the time of this study. Twelve participants (44.4%) reported that they were previously participating in community-based activities.

Breast cancer (*n* = 12, 44.4%) was the most common type of cancer diagnosis, both overall and among female participants, while bowel cancer (*n* = 3, 11%) was the most frequently observed cancer among male participants. The majority of participants (*n* = 18, 66.6%) received their cancer diagnosis up to three years prior to participation in the study (Table 1).

### 3.3. Health Outcomes

Twenty-seven participants completed outcome measures at baseline and follow-up. All measures showed improvements in the total and category scores following social prescribing except for the EORTC “Symptom Score” category (Table 2). Statistically significant improvements were observed in the FAI total score (*p* = 0.01) and the FAI category “leisure/work” (*p* = 0.001).

There was a statistically significant improvement in the “Depression” scale of the HADS following participation in social prescribing (*p* = 0.025), and the median anxiety score reduced from above the cut-off score for the case level of anxiety to below the cut-off score following participation in social prescribing (Table 2). Upon examining the fatigue-related outcomes of social prescribing, the category of “Distress caused by Fatigue” reduced significantly (*p* = 0.045).

The functional category of EORTC QLQ-C30 includes physical, emotional, role, cognitive and social functioning. This category improved significantly following participation in social prescribing (Table 3).

### 3.4. Qualitative Findings

All twenty-seven study participants completed semi-structured interviews exploring their perspectives on the impact of social prescribing and activity engagement on their health and their experiences of attending social prescribing. Following data analysis, four themes were identified:

➢ Barriers to activity engagement prior to attending social prescribing;
➢ Outcomes of being connected to activities or services;
➢ Barriers to participation in social prescribed activities or services;
➢ Experiences of attending social prescribing.

#### 3.4.1. Barriers to Activity Engagement Prior to Attending Social Prescribing

Participants discussed the impact of cancer-related symptoms on their ability to participate in activities. Symptoms included fatigue, cognitive challenges and anxiety.

The majority of participants identified fatigue as a cancer-related symptom that impacted on engaging in activities:

*“The fatigue was a big thing. Definitely it’s been like one of the most prominent things in my life physically since my diagnosis.”*

(P13)

Some participants also identified difficulty sleeping and how that impacted on their fatigue:

*“And then insomnia as well that I suffer from doesn’t help with the fatigue”*

(P19)

Some participants identified the impact of cancer on their mental health:

*“I have issues with depression and anxiety. Whether that’s from the cancer I don’t know but I have no doubt it has a certain role to play in it.”*

(P13)

*“The fear, oh my god, the terror of it coming back. That stayed with me a long, long time after the treatment was finished.”*

(P20)

One of the interview participants identified body image as a factor influencing activity engagement:

*“I’d say it’s probably mostly for women but when you have a bit of hair on your head you feel like you can attend activities. Even if it’s not too much, you feel more able to start becoming yourself again in that sense.”*

(P6)

Some participants also stated that they did not want to attend cancer-specific services as they did not want to be identified as a person with cancer. However, they did not consider social prescribing as a cancer-specific service:

*“The mere fact of going in through the door* [name of cancer centre] *said to me, ‘oh yeah, you have cancer, you know that don’t you?’ Now here* [social prescribing service]*, nobody said that.”*

(P20)

#### 3.4.2. Outcomes of Being Connected to Activities or Services

Participants identified improved physical health following attendance at activities they accessed following support from the link worker:

*“I have more energy. And when I’m finished these classes I can go home and I can do a lot more work in the house, you know what I mean? You’re more energetic.”*

(P3)

Improvements in mental health were also identified:

*“I think my mental health is definitely improved since, I don’t feel as anxious and as stressed as I possibly did—no, I definitely don’t.”*

(P17)

Study participants also identified increased opportunities for social interaction:

*“I’m definitely meeting new people and strangers who I’ve never met before and I really like that. I just think meeting new people is so fun”*

(P6)

Other benefits included improved motivation and how engaging in activities provided a structure to daily routines:

*“It’s kind of a reference point and then you can look at the calendar and say, ‘Okay I have this and then I have that.’ And then things can sort of hang on from there. I have some routine and so I know what’s going to happen”*

(P9)

Some participants joined additional activities following their initial attendance at the activity the link worker identified:

*“By attending one thing then I thought I can do something else you know? It’d be nice to come up here and take up something else.”*

(P10)

#### 3.4.3. Barriers to Participation in Social Prescribed Activities

Barriers to attending community-based activities included lack of places available in preferred activities and difficulty accessing activities due to reduced mobility. One participant wanted to attend yoga near her home, but the class was consistently full. She therefore participated in an online yoga class but reported that it did not have the social impact of attending a face-to-face class:

*“I enjoyed the yoga at home and feel that it benefited me a little bit. But it wasn’t like a class setting, so I wasn’t meeting anybody which was what I was looking forward to was meeting new people.”*

(P20)

Limited mobility combined with poor weather restricted another participant from attending his preferred activities:

*“The weather affects me. And as I say, my walking is not great and I don’t like walking into the breeze going up to the community centre. Because of that I have to walk into town to get a bus and then when I get off the bus, I still have to walk. So I decided I wouldn’t go.”*

(P4)

#### 3.4.4. Experiences of Attending a Social Prescribing Service

Overall, participants spoke positively of their experience of social prescribing. They discussed support received from the link worker:

*“I was coming here and knew it was for cancer, so I knew I could talk about it without any barriers. I knew I wouldn’t put fear into you because you are dealing with it and I could talk about it no problem. Believe it or not I felt a big lump off my shoulders going out after the meeting to be able to talk about it.”*

(P7)

Some participants valued the support from having the link worker accompany them to their chosen activity:

*“But she has a lovely manner and she met me there when I went because I was a bit insecure the first day going because I didn’t know what to expect.”*

(P11)

Receiving continuing information was also valued by participants:

*“I received emails and different things from the link worker like new courses or new activities that she came across. So I definitely knew that the support was always there and if I had an issue, or if I needed help working on something, that I could just give her a call or let her know what I was doing, things like that.”*

(P13)

Another participant identified the benefit of the link worker identifying local activities:

*“You just don’t realise what’s going on in your area until someone else researches it for you”*

(P10)

## 4. Discussion

The purpose of this study was to examine the feasibility of social prescribing for individuals with cancer. The recruitment rate for this study was close to 33%. A recent review of recruitment rates to intervention studies in cancer survivorship reported a median rate of 38% indicating an acceptable recruitment for this current study [27]. The adherence and retention rates for this current study were 62.7% for both, which indicates high acceptability of the intervention. This was supported in the qualitative findings with participants reporting a range of health-related benefits from participating in social prescribing. These findings indicate that a community-based social prescribing service is feasible for individuals living with and beyond cancer.

The majority of participants in this study were women with breast cancer. A recent review of self-management studies identified breast cancer survivors as the main participants in self-management interventions [28]. Therefore, future studies of social prescribing should consider alternative methods to recruit more men and individuals with other types of cancer. The majority of individuals who agreed to participate in this study were up to three years post-cancer diagnosis. This would indicate that this is potentially the time range at which cancer survivors are ready to re-engage in community-based activities to support their physical, mental and social health. The small numbers in this study did not enable comparisons between individuals at different times from cancer diagnosis. A larger study is needed to explore these differences.

Twenty-seven people met with a social prescribing link worker and attended a range of community-based activities. Following attendance at their activities of choice, participants had statistically significant improvements in how frequently they engaged in activities, specifically work and leisure activities. They also had statistically significant improvements in depression and fatigue-related distress following involvement in the service. As this is a feasibility study, it cannot establish whether social prescribing or some other factor resulted in these significant improvements. Therefore, the findings of this study support the need for a more rigorous study that can determine the extent to which social prescribing can improve the health and well-being of individuals living with and beyond cancer.

This feasibility study also demonstrates the acceptability of a social prescribing service to individuals living with and beyond cancer. Participants reported improved mental, physical and social health as a result of attending community-based activities which they accessed with support from the link worker in the social prescribing service. They also experienced increased motivation to participate in activities and discussed how attending community-based activities provided them with a structure and routine to their day. Individuals living with and beyond cancer valued the support from a link worker in identifying and accompanying them to their preferred activities. They also identified the benefits of attending non-cancer-specific services.

### Impact on Physical and Mental Health

Cancer and cancer treatment result in a range of mental and physical health difficulties [29]. In this current study, participants reported experiencing cancer-related difficulties including fatigue, cognitive challenges, low confidence and sleep disturbance. Previous research has described the impact these symptoms have on daily activity participation, including the ability to socialise and engage in leisure activities [30]. Following their participation in social prescribing, participants’ frequency in which they engaged in social and leisure-related activities also increased significantly. Some participants reported that through attending one activity in their local community centre, they became more aware of other activities offered in the centre and signed up for these activities independently of input from the link worker. Perhaps social prescribing gave participants the motivation and confidence to explore other activities without the support of a link worker. Increased activity participation aligns with other studies that have examined the impact of social prescribing [7,8,31]. However, a more rigorous study, such as a randomised control trial, is required to provide definitive evidence of this outcome of social prescribing.

Fatigue is a persistent and debilitating cancer-related symptom which can endure for many years following cancer treatment [32]. It interferes with many activities of daily living and is reported as a symptom that prevents some cancer survivors from returning to work following successful completion of treatment and negatively impacts on quality of life [33]. In cancer patients, disturbed sleep, which contributes to fatigue, is rated the second most bothersome symptom based on cancer type and treatment status [34]. Some of the participants in this current study identified difficulty sleeping which resolved following their participation in community activities. It is possible that improved sleep patterns contributed towards reducing fatigue as participants reported significantly less fatigue-related distress. The participants in this study engaged in a wide range of physical-based activities such as dance and yoga which could have increased fitness levels and contributed to reduced fatigue. Previous research reported that fatigue was improved through engagement in physical activities [32].

In their interviews, some participants identified experiencing anxiety following treatment, citing one reason for this being fear of cancer recurrence. Following participation in social prescribing, participants described how their mood improved. Other studies have also reported improved mood following participation in social prescribing [7]. The causal mechanism for improved mood in social prescribing is as yet unclear. Some suggestions include that improved mood is as a result of person-centred interactions between an individual attending the service and their link worker, as a result of the individual attending targeted activities that impact on mood or perhaps both [35]. Further research on mechanisms of change following social prescribing interventions is required to explore this.

In a recent systematic review of social prescribing outcomes, Pescheny et al. [36] reported reduced social isolation as a frequent positive outcome. In this current study, participants referred to increased social interaction as a reason to participate in social prescribing rather than completing activities alone. Social prescribing offered participants a chance to meet people while also participating in health-related activities. Previous studies have identified how reduced confidence impacted on re-engaging in social activities following completion of cancer treatment [34].

Cancer survivors identified the link worker as a key factor in their experience of social prescribing. The role of the link worker has been highlighted repeatedly as a key mechanism to promote positive change [31,37]. Participants described the link worker as supportive, accommodating and offering reassurance. The link worker offered emotional support and reassurance to study participants which has been found to be important in supporting service users who lack self-esteem and experience anxiety about engaging in the process of social prescribing [38].

Due to difficulty accessing their chosen activities, some participants who had started attending local activities discontinued following one or two attendances. One participant was unable to secure a place in a yoga class due to lack of availability. Another participant had mobility issues that prevented him from attending his local community centre. Successful social prescribing is dependent on the availability of adequate and suitable activities and services in local communities to ensure individuals can participate in their activities of choice. Wildman et al. [38], reported that gaps in activities and supports were due to lack of affordable and accessible services. Many community-based services in Ireland are provided by the Voluntary and Community Sector, who rely, to a large extent, on fund-raising and charitable donations for delivering and developing services. A steady funding stream is critical to ensure access to high-quality and sustainable services to which link workers can refer individuals [39].

A strength of this study is that it is one of only a few studies to examine the feasibility of social prescribing for individuals living with cancer. Social prescribing is considered a complex intervention that requires different data collection methods to examine its impact on health and well-being and identify critical components of this intervention [14]. Therefore, the mixed methods design used in this study provided rigorous data to progress this study to a pilot randomised control trial of social prescribing. A limitation of this study was that the majority of participants were female. Although previous studies have identified a reluctance of men to avail of community-based supports, in order to fully evaluate social prescribing for individuals with cancer, future studies will need to focus on recruitment strategies to ensure increased male participation.

## 5. Conclusions

The purpose of this study was to examine the feasibility and acceptability of social prescribing for cancer survivors. Social prescribing is considered a non-medical community-based support to improve the health and well-being of individuals with chronic health conditions. Although much of the research to date has focused on the impact of social prescribing for people with mental health difficulties, there is little research exploring the feasibility of this service for cancer survivors.

This study demonstrated that social prescribing is a feasible and acceptable intervention for cancer survivors. Improvements were observed in participants’ physical, mental and social health following their meetings with a link worker and attending local community-based activities, indicating the potential benefit of social prescribing for cancer survivors. The findings of this study support further investigation of social prescribing for individuals living with and beyond cancer.

## Figures and Tables

**Table 1:** Years since primary cancer diagnosis.

	Number of Participants	Frequency
**<1 year**	8	29.7%
**1–3 years**	10	37%
**>3 years**	9	33.3%

**Table 2:** Median scores of the FAI ^1^, HADS ^2^ and MAF ^3^ T0 and T1.

Measure	Time 1 (T0) Median Score (IQR)	Time 2 (T1) SP Scores (IQR)	*p*-Value
**FAI ^1^**			
Domestic	13 (4)	14 (3)	0.317
Leisure/work	8 (2)	10 (3)	0.001 *
Outdoors	11 (2)	11 (2)	0.282
Total FAI	32 (6)	35 (6)	0.01 *
**HADS ^2^**			
Anxiety	8 (8)	5 (6)	0.10
Depression	5 (5)	2 (4)	0.025 *
**MAF ^3^**			
Fatigue severity	5 (3.5)	4 (4)	0.11
Fatigue-related distress	4 (5)	2 (3)	0.045 *
Interference of fatigue with ADL	3.11 (4)	2 (2.4)	0.21
Global Fatigue Index	24.56 (19.8)	17.89 (16.4)	0.12

^1^ FAI: Frenchay Activities Index; ^2^ HADS: Hospital and Depression Scale; ^3^ MAF Multidimensional Assessment of Fatigue. * Indicates statistical significance.

**Table 3:** Category scores of EORTC QLQ-C30 at T0 and T1.

	Baseline (T0) Median (IQR) (n = 27)	Post-Intervention (T1) Median (IQR) (n = 27)	Change in Median Scores	*p*-Value
**Global Health Status**	66.67 (33.33)	75 (16.66)	8.33	0.5
**Functional Status**	75 (23.66)	90.33 (15.33)	15.33	**<0.001**
**Symptoms**	16.05 (17.91)	15.43 (12.96)	−0.62	0.431
**EORTC Summary Scores**	80.32 (20.16)	85.87 (14.92)	7.26	0.18

## Data Availability

The data presented in this study are available on reasonable request from the corresponding author.

## Supplementary Materials

The following supporting information can be downloaded at https://www.mdpi.com/article/10.3390/curroncol32030129/s1, File S1. Interview guide for cancer survivors on acceptability of social prescribing for cancer survivors.
