# Supplementary material for: Feasibility and Acceptability of Social Prescribing for Cancer Survivors
Source: Curr Oncol. 2025 Feb 25;32(3):129. doi: 10.3390/curroncol32030129 (PMC11940869; doi:10.3390/curroncol32030129)
Supplement: Supplementary file 1 [file curroncol-32-00129-s001.zip › curroncol-3449942-supplementary.pdf]

An exploration of the impact of a social prescribing service for cancer survivors

Interview Schedule

- Did the social prescribing link worker help you to identify any activities and/or services in your local community in which you wanted to participate?

- Did you attend the activities and/or services identified by the link worker?
    - If yes, what activities and/or services did you attend?
    - How did you access the activities and/or services
    - Did you enjoy activities and/or services?

- If you did not attend the activities and/or services identified by the link worker, what were some of the reasons for not attending?

- In what way did attending the activities and/or services identified by the link worker make any difference to your:
    - physical health
    - mental health
    - social health
    - self-confidence

- Have you started doing other activities and/or services in your local community as a result of attending social prescribing?
    - If yes, what are these activities and where are they based?
    - If no, why not?

- In your experience, is social prescribing helpful for individuals with cancer?

- Have you any recommendations on how to improve and/or change this social prescribing service for cancer survivors?
